# Supplementary material for: Crystal structure of a natural light-gated anion channelrhodopsin
Source: eLife. 2019 Jan 7;8:e41741. doi: 10.7554/eLife.41741 (PMC6336409; doi:10.7554/eLife.41741)
Supplement: Transparent reporting form [file elife-41741-transrepform.docx]

***eLife’s* transparent reporting form**

We encourage authors to provide detailed information *within their submission* to facilitate the interpretation and replication of experiments. Authors can upload supporting documentation to indicate the use of appropriate reporting guidelines for health-related research (see [EQUATOR Network](http://www.equator-network.org/%20)), life science research (see the [BioSharing Information Resource](https://biosharing.org/)), or the [ARRIVE guidelines](http://www.plosbiology.org/article/info:doi/10.1371/journal.pbio.1000412) for reporting work involving animal research. Where applicable, authors should refer to any relevant reporting standards documents in this form.

If you have any questions, please consult our Journal Policies and/or contact us: [editorial@elifesciences.org](mailto:editorial@elifesciences.org).

**Sample-size estimation**

- You should state whether an appropriate sample size was computed when the study was being designed
- You should state the statistical method of sample size computation and any required assumptions
- If no explicit power analysis was used, you should describe how you decided what sample (replicate) size (number) to use

Please outline where this information can be found within the submission (e.g., sections or figure legends), or explain why this information doesn’t apply to your submission:

A statement about a sample size determination is included in the Material and Methods section: “Sample size was estimated from previous experience and published work on a similar subject, as recommended by the NIH guidelines (Dell et al. 2002).”

**Replicates**

- You should report how often each experiment was performed
- You should include a definition of biological versus technical replication
- The data obtained should be provided and sufficient information should be provided to indicate the number of independent biological and/or technical replicates
- If you encountered any outliers, you should describe how these were handled
- Criteria for exclusion/inclusion of data should be clearly stated
- High-throughput sequence data should be uploaded before submission, with a private link for reviewers provided (these are available from both GEO and ArrayExpress)

Please outline where this information can be found within the submission (e.g., sections or figure legends), or explain why this information doesn’t apply to your submission:

The information about replicates appears in the Materials and Methods section as following: “Transfection with each tested mutant variant was repeated in at least three different batches of culture, and the results obtained in cells from all batches were pooled. <…> Individual transfected HEK293 cells were selected for patching by inspecting their tag fluorescence; non-fluorescent cells were excluded. Cells in which we could not establish a gigaohm seal were automatically discarded from measurements. Data were also excluded if a gigaohm seal was lost during recording. Current traces recorded from the same cells upon repetitive light stimulation were considered as technical replicates; results obtained from different individual cells were considered as biological replicates. In experiments with laser excitation, 10 technical replicates were averaged to yield a single mean trace for each cell; in experiments with continuous light pulses, a single trace was recorded in each cell. The baseline measured before illumination was subtracted using Clampfit software (a subroutine of pClamp). The same software was used to measure the peak current amplitude with a cursor. The raw data obtained in individual cells are shown as open diamonds and listed in the corresponding source data tables. No outliers were excluded from calculating the mean values.”

**Statistical reporting**

- Statistical analysis methods should be described and justified
- Raw data should be presented in figures whenever informative to do so (typically when N per group is less than 10)
- For each experiment, you should identify the statistical tests used, exact values of N, definitions of center, methods of multiple test correction, and dispersion and precision measures (e.g., mean, median, SD, SEM, confidence intervals; and, for the major substantive results, a measure of effect size (e.g., Pearson's r, Cohen's d)
- Report exact p-values wherever possible alongside the summary statistics and 95% confidence intervals. These should be reported for all key questions and not only when the p-value is less than 0.05.

Please outline where this information can be found within the submission (e.g., sections or figure legends), or explain why this information doesn’t apply to your submission:

The information about statistical testing appears in Material and Methods section as following: “Normality of the data was not assumed, and therefore non-parametric statistical tests were used as implemented in OriginPro 2016 software; P values > 0.05 were considered not significant. The results of statistical hypothesis testing are shown in Tables 2-4. When no specific statistical hypothesis was tested, descriptive statistical analysis was applied.”

(For large datasets, or papers with a very large number of statistical tests, you may upload a single table file with tests, Ns, etc., with reference to sections in the manuscript.)

**Group allocation**

- Indicate how samples were allocated into experimental groups (in the case of clinical studies, please specify allocation to treatment method); if randomization was used, please also state if restricted randomization was applied
- Indicate if masking was used during group allocation, data collection and/or data analysis

Please outline where this information can be found within the submission (e.g., sections or figure legends), or explain why this information doesn’t apply to your submission:

The information about group allocation appears in Material and Methods section as following: “Batches of culture were randomly allocated for transfection with a specific mutant; no masking (blinding) was used.”

**Additional data files (“source data”)**

- We encourage you to upload relevant additional data files, such as numerical data that are represented as a graph in a figure, or as a summary table
- Where provided, these should be in the most useful format, and they can be uploaded as “Source data” files linked to a main figure or table
- Include model definition files including the full list of parameters used
- Include code used for data analysis (e.g., R, MatLab)
- Avoid stating that data files are “available upon request”

Please indicate the figures or tables for which source data files have been provided:

The source data tables are provided for Figs. 3G, 4D, 3S4, 3S6, 3S9A and 3S9B.
